# Addressing oil price changes through business profitability in oil and gas industry in the United Kingdom

**DOI:** 10.1371/journal.pone.0199100

**Published:** 2018-06-21

**Authors:** Sorana Vătavu, Oana-Ramona Lobonț, Iulia Para, Andrei Pelin

**Affiliations:** 1 Department of Finance, Faculty of Economics and Business Administration, West University of Timisoara, Timisoara, Romania; 2 Department of Marketing and International Business Relations, Faculty of Economics and Business Administration, West University of Timisoara, Timisoara, Romania; 3 Department of Management, Faculty of Economics and Business Administration, West University of Timisoara, Timisoara, Romania; Central South University, CHINA

## Abstract

In this paper, we investigate how crude oil price and volume traded affected the profitability of oil and gas companies in the United Kingdom (UK) since the financial crisis started in 2008. The study benefit from insights of the financial statements, to develop a model that focuses on how changes in oil price impact corporate performance. In order to observe the financial indicators that influence the performance, as well as the effects that changes in oil prices and demand of crude oil have on the profitability of oil and gas companies, we apply comparative regression analysis, including the generalised method of moments estimation technique for panel data set. The sample is consisting of 31 oil and gas companies in the UK, and the period analysed is 2006–2014. Results show that profitable oil and gas companies managed to face the drop in oil price and recover, characterized by significant cash flows and stock turnover, efficient use of assets, and high solvency rates. Although the oil price and volume traded do not significantly affect profitability and other financial ratios, if the oil price continues to decrease, it would permanently alter both the UK economy and oil and gas companies. In order to survive, companies make drastic cuts and defer essential investments, often at the long-term expense of asset performance. This study is important in a world where the energy consumption steadily grew over time. However, the renewable energy is cheaper and more environmentally friendly, and thus, countries where oil and gas industry is one of the most popular sectors face an economic decline. These results could be useful for investors, managers or decision makers, reclaiming strategic decisions in the current uncertain and volatile environment.

## Introduction

This paper intends to investigate the relationship between oil price and corporate performance in a European country where the energy industry is one of the essential components of the economy. The UK offshore oil and gas industry used to contribute to 2.5% of GDP before 2010, but in 2015 it only counted for 0.8%. Fiscal revenues declined from approximately £7 billion before 2010 to 2 billion in 2015 and to almost zero in 2016. Also, jobs continue to be made redundant, leaving a high unemployment rate. Although it is intuitive for the performance of oil and gas companies to be strongly related to oil prices, there are few papers to study this relationship, while many focused on the impact of oil price shocks on a macroeconomic level. This paper aims to observe to what extent were the oil and gas companies affected after the financial crisis began, and since the oil price dropped, to review corporate performance in times of high volatility and increased financial risks.

The world is dependent on energy, and thus consumption steadily grew over the last decades. Large economies such as the United States and China became the most significant oil consumers. China doubled its energy consumption over the first decade of the 21^st^ century and accounted for approximately 60% of the world’s growth in oil consumption over the last decade. The European Union is situated between the US and China in the list of countries by oil consumption, relying on net imports for 89% of the oil products consumed. The major producer of crude oil in the EU is the United Kingdom, followed by Denmark, Italy, and Romania (the last three countries produced less than half of the UK primary production of crude oil in 2015). The financial markets developed over the oil-related funds all over the world and thus many investors pay great attention to oil price fluctuations, while oil and gas companies increased the number of mutual and exchange-traded funds.

Nowadays, even the most profitable and stable companies suffer from the crisis threatening the oil and gas industry. After an extended period of high oil prices, the industry is now facing an extended period of low prices, amplified by a decrease in the operational efficiency of oil and gas companies due to higher extraction costs. On the contrary, economical fossil fuels allow higher standards of living for the population and thus, economic growth on this side, but significant costs regarding pollution or oil spills.

The oil and gas industry was always volatile, facing boom-and-boost cycles. Thereby, companies tend to invest in assets and raise employees number in order to grow during prosperous periods, while making drastic cuts and dropping profitable projects, to minimize costs during downturns.

Economic factors also put pressure on this industry, while digital technologies offer new tools that simplify operational processes and increase production. However, these are costly and require intelligent investing. Therefore, oil and gas companies are forced to create strategies for sustainable change, bringing stability, improving the primary performance metrics and operational excellence through booms and busts.

The main contribution of this paper comes from the analysis perspective, including besides the financial information, data reflecting the dynamics of the oil industry to observe the corporate performance, which is not only critical to companies operating in this field, but also to oil-dependent countries such as the UK. This research was undertaken to analyse the profitability of oil and gas companies in the UK, where unemployment rate and losses steadily grew over the last years due to the decrease in oil price, affecting the economy at different levels. The profitability is analysed based on assets efficiency, liquidity, and solvency, concerning crude oil prices. As the literature was primarily focused on the relationship between oil prices and stock markets, our study analyses the performance of oil and gas companies over the financial crisis and the periods of falling oil prices, reviewing their operational activity, and their ability to generate profits. Therefore, we seek to fill the gap in the present literature, developing the research into adjacent points of interest that have not been debated recently. In addition, the study is generalised to the corporate performance in the oil and gas industry, in the UK, while previous studies focused on the effects that oil prices have on individual companies.

The following section reviews the literature related to oil and gas industry performance over time. The third section presents the data used and the methodology. Section 4 describes the empirical results obtained through different stages of analysis. Firstly, correlations between all variables are discussed, followed by a presentation of the baseline regression results. Secondly, a non-linear analysis has been employed in order to capture the structural breaks in oil and gas companies performance, financial ratios, and crude oil prices. These various stages of the study were also intended to test the results robustness. Section 5 concludes.

## Literature review

The financial crisis represented a turning point for some major national oil companies around the world. For example, although the competition for resources increased, in China, it was discovered that some of the world’s biggest oil companies became the most efficient regarding production, mostly based on investment opportunities, expansions, and international cooperation agreements. National Chinese oil companies were also an advantage because of the production scale, and due to domestic macroeconomic regulations focused on protecting the monopolistic organisations and based on the gradual increase in the domestic oil demand. However, it was admitted that there is an unobserved heterogeneity with parameters related to the centrally planned economy or institutional environment, which may influence the analysis and cause unbiased results in oil companies efficiency on the subject of production [1]. These make the oil industry in China react differently to the financial crisis compared to other market economies. Other researchers agreed on the fact that unobserved heterogeneity among oil industry may produce biased efficiency in results, and factors related to market reforms and government policies, economic development or social environment may induce different strategies and operational activities among oil companies from different countries [2].

Studies also proved that corporate governance has an essential impact on oil companies’ profitability and performance. Besides, ownership concentration also has a strong influence on firm performance, showing a nonlinear relationship. Studies prove the fact that, on a risk-adjusted basis, companies with strong shareholders rights outperform, proving that good governance has a positive impact on corporate performance [3,4]. Furthermore, it seems that companies with private ownership are more efficient than state ownership ones [5]. Previous studies focused on the relationship between oil prices and the share prices of oil and gas companies. For example, in the US, it was showed that returns are strongly affected by the shocks in the oil market and the economic policy uncertainty, while the shocks on demand have a positive effect on returns [6]. Although in this case results are more evident over the long run (after a period of 60 months), other researchers found through an unrestricted VAR model a significant positive impact of oil price shocks on stock returns, over the short-run [7]. For the oil and gas companies in the UK, the oil price shocks seem to have both, negative and positive effects on stock prices [8]. More specifically, the study shows through wavelet analysis that over the short term risks and oil price have weak effects on oil and gas industry investors, who can still diversify their portfolios’, but risks become more important for long-term investors. Actually, for them, previous oil price information can be very useful to improve the forecasting quality of future stock prices.

The fact that changes in oil price have a positive effect on stock returns, and an increase in oil prices does not depress the demand of oil to a large extent, shows that oil and gas companies easily pass oil price increases on to customers. Depending on the corporate culture, managers and shareholders are focused on maximising companies’ operational efficiency over long-term through constant returns and gradual growth. Oil companies should also have an increased level of control and transparency in order to safeguard investor’s interests and bank officials offering resources for new investments [9]. Moreover, a campaign requiring transparency in company payments, government revenues and expenditures, and licensing procedures, was launched in 2002 in order to strengthen corporate governance in extractive sectors [10]. This campaign was entitled the “Extractive Industries Transparency Initiative”—EITI and was established by the UK prime minister, and implemented in 39 countries through a government-driven process. It was shown that the countries implementing EITI do not outperform others, due to corruption, stakeholders’ ignorance to implications, especially in the case of state-owned companies, and strong dependence on civil society [11].

The liberalization of capital flows increased the competition in globalized markets, although the EU members tried to reduce the disparities between the integrated European areas. In the recent context, companies reach competitiveness through innovation and research, focusing on increasing productivity, but also supporting sustainable economic growth and citizens’ quality of life [12]. Research on the recent financial crisis indicated that oil prices affect stock prices in all stock markets, although the supply and demand have not been affected to that extent. Therefore, oil companies were blamed for price gauging, as studies showed that commodity prices mainly drive their performance. Firms claim that their profits have been reduced due to crude oil price volatility, after investing billions of dollars and paying high taxes. Results are different, depending on the periods and countries analysed, showing that oil prices may have a weak impact on non-commodity based stock markets (like in the UK or Japan). For example, in North America, crude oil prices have a positive impact on oil and gas sector profitability but, through the recent financial crisis, the relationship became an indirect one, with a negative influence from oil prices to corporate performance. However, other crisis such as the Asian one or the 9/11 did not influence firm performance significantly [13]. From a comparison between risk factors in developed and developing countries, it was shown that oil and gas industry responds more strongly to the volatility in oil prices in developed countries. Moreover, the returns in this industry tend to be asymmetric, as increases in oil price have a more significant impact than price drops [14].

In developed countries, oil and gas companies have robust command-and-control regulations, while the lack of control, corruption and weak enforcement of legislation penalise returns and productivity in developing economies. This is a strong reason for companies to use their know-how and resources to change the situation in developing countries and in economically disadvantaged communities, where selling off natural resources causes poverty and low wages [15,16]. Although companies need intelligent investing that is costly, this ensures a continuous generation of cash and higher operating profits. For a reduced bankruptcy risk, shareholders should agree on increasing equity, or even changing debt into shared capital [17]. Moreover, oil and gas companies are now required to take into consideration environmental friendly strategies, as this is one the crucial competitive advantages proved to have a positive influence on firm performance and profitability [18].

The aim of this study contributes significantly to this field of research as it focuses on the question related to what extent the oil price may drop until it would permanently affect oil and gas companies performance and the UK economy. Considering that the relationship between the stock prices and oil price was analysed over time, our study will focus more on companies’ financial performance rather than on shareholders’ returns, reviewing their operational activity as well as the level of the taxes paid, based on oil consumption and its prices. The period analysed is suitable to reveal if there is an effect over the short or long-term period, and the sample is adequate to consider the results specific to the oil and gas industry in the UK. Therefore, the study is more general than previous works that considered influences on individual companies, and thus a limited number of companies.

## Data and methodology

### Data and variables

This study evaluates the profitability of oil and gas companies in the UK, based on different financial indicators, but also trying to capture to what extent profitability was affected by the changes in crude oil prices. To obtain more information on the profitability of oil and gas companies, the analysis will include besides the evidence on oil prices and corporate performance, financial information related to the companies, such as assets efficiency, liquidity, solvency or the level of taxes paid. Considering the recent steady period of low oil prices, and the switch towards renewable energies, it is expected for oil and gas companies profitability to drop. Therefore, it is essential to overview how efficiently they operate and the financial risks undertaken.

The analysis is based on annual data, over the period 2006–2014. The following financial indicators and ratios used in this study were collected from Amadeus database, which offers financial information on companies across Europe: return on equity (ROE), cash flow over operating revenue (CF/OpRev), current ratio (CurrRat), Asset turnover (AssTn), solvency ratio (SolvRat), stock turnover (StockTn), and finally, taxation, computed as the natural logarithm of the value paid by companies every year as taxes (lTax). The data related to the oil industry (crude oil price, as $/barrel; the volume of oil traded every month, in millions of barrels) was obtained from the website Investing UK. It was then used to compute three control variables, with identical annual values regardless of the company considered: OilPrice, as the annual average of the crude oil price, OilVolume, as the annual average amount of oil traded, and a dummy variable (dummyOilPrice) which takes the value 1 when the annual average oil price drops compared to the previous year’s average, or 0 if it increases.

In order to obtain robust results, we collected data for a limited sample of 31 oil and gas companies, due to the fact that many companies in this industry had missing data from their balance sheets, profit and loss accounts, and global ratios over the period considered. The data collected were analysed with STATA, a statistical software package commonly used in the economics field, to observe statistical analysis, graphics and regressions. All the results further presented and those from the “Results and analysis” section represent authors’ computations in STATA.

Table 1 below presents the descriptive statistics of the variables considered in this research.

**Table 1:** Descriptive statistics.

Variable	Obs	Mean	Std. Dev.	Min	Max
**ROE** (%)	279	27.6777	38.2054	-119.53	310.7
**CF/OpRev**	279	35.5832	22.1877	-30.88	97.81
**CurrRat**	279	2.4939	3.6119	0.08	37.59
**AssTn**	279	1.4560	2.2535	0.07	17.84
**SolvRat**	279	46.6986	21.6390	8.56	92.86
**StockTn**	279	44.7989	66.3885	2.12	724.5
**lTax**	250	11.8915	1.9748	6.5356	16.7504
**OilPrice** ($/barrel)	279	84.9207	12.9505	63.9233	98.5833
**OilVolume** (barrel)	279	5.1808	1.1365	2.5033	6.6125

Return on equity is on average 27.67%, which indicates high profitability of oil and gas companies in relation to the book value of shareholders’ equity. The cash flow over operating revenue ratio also proves that oil and gas companies are profitable, showing investors and shareholders that most oil and gas companies can generate consistent cash from their sales. With an average current ratio of 2.5, companies analysed can pay off their short-term liabilities based on their current assets, which is another proof that they are financially stable. Based on the average asset turnover of 1.45, the oil and gas companies are efficient regarding their assets, as they are generating sufficient sales revenue. The average solvency ratio of 46.7, the companies observed also prove that they can meet their long-term obligations, sustaining their operations indefinitely. The stock turnover is very high, approximately 45, showing that the oil and gas companies’ inventory is used or sold quickly.

Tax variable shows an average of approximately $150 million (lTax value of 11.89) paid annually by the oil and gas companies in the UK. For this variable, some data is missing as companies rarely registered negative values on Taxation, and therefore these values could not be computed based on the logarithm.

Over the period analysed, from 2006 to 2014, the average price of the crude oil was $84.92, while the volume of the oil traded was of approximately 5 barrels/month, increasing very much from the first year until the last ones.

Profitability will be considered as a function of multiple indicators, some internal and other exogenous to the companies, as presented in the following equation:

$$Profitability=f(\frac{cash\;flow}{operating\;revenue},current\;ratio,asset\;turnover,solvency\;ratio,stock\;turnover,taxation)$$ (1)

In order to test the results robustness, another model will be tested, based on the previous equation where the three control variables, related to the oil price and volume traded, are added:

Profitability=f(cashflowoperatingrevenue,currentratio,assetturnover,solvencyratio,stockturnover,taxation,crudeoilprice,crudeoilvolumetraded,dummyoilpricedecrease) (2)

The linear model of the profitability function can be expressed by the following two equations:

$${ROE}_{it}=\alpha_{i}+\beta_{1}{\frac{CF}{OpRev}}_{it}+\beta_{2}{CurrRat}_{it}+\beta_{3}{AssTn}_{it}+\beta_{4}{SolvRat}_{it}+\beta_{5}{StockTn}_{it}+\beta_{6}{lTax}_{it}+\varepsilon_{it},$$ (3)

$${ROE}_{it}=\alpha_{i}+\beta_{1}{\frac{CF}{OpRev}}_{it}+\beta_{2}{CurrRat}_{it}+\beta_{3}{AssTn}_{it}+\beta_{4}{SolvRat}_{it}+\beta_{5}{StockTn}_{it}+\beta_{6}{lTax}_{it}+\beta_{7}{OilPrice}_{t}+\beta_{8}{OilVolume}_{it}+\beta_{9}{dummyOilPrice}_{t}+\varepsilon_{it,}$$ (4)

where α_i_ represents the unknown intercept of each of the 31 companies included in the sample (i = 1…31), t is the year analysed (t = 2006…2014), the coefficients associated to each explanatory variable are the βs, and the error term is ε_it_.

### Methodology

The relationship between oil price and returns was mostly studied through vector autoregressive—VAR or structural vector autoregressive models—SVAR [6,7]. Within VAR models, the business cycle is defined by output movements associated with shocks. These shocks are assumed to be without long-run effects on output, but previous studies, as well as this one, show that there is a significant lag between the changes in oil prices and their impact on company returns. In addition, in these models, the exogenous oil price changes do not make a distinction between oil and demand shocks. Moreover, it is very difficult to find totally exogenous variable especially in micro-economic models, where all the indicators are interdependent, and thus endogenous to some extent. Contrary to the previous studies based on VAR or SVAR results, which referred to individual oil and gas companies, we aim to observe the overall oil and gas industry, based on a sample of 31 companies. We will use both, static and dynamic models, developing the analysis through multiple regression models. The final step in our analysis will be estimating an instrumental variable panel VAR, which offers the responses of the variables used to an exogenous shock, after controlling for time-invariant characteristics of individual companies. As long as results from previous research seem robust for sub-periods [7], we expect a nonlinear relationship between oil price and corporate performance, and thus, will use nonlinear regression models as well.

First of all, this analysis reveals, through descriptive statistics, the dynamics of the variables observed, capturing their primary influences over profitability. In order to test whether or not the explanatory variables have a significant impact on return on equity, a comparison between different regression models will be realised: the first one considered is Pooled Ordinary Least Square (OLS) model, followed by Fixed Effect (FE) and Random Effect (RE) models. All will be regressed on the overall panel of oil and gas companies, for the nine-year period. One of the advantages of panel data analysis is accounting for individual heterogeneity while controlling for unobserved differences in corporate practices through time. The Hausman Test will be used to reflect the accuracy of fixed effect and random effect models. Therefore, if there are specific companies characteristics which would influence other variables, the fixed effect model is more appropriate. On the contrary, the random effect model is useful for samples in which variation across entities are random and uncorrelated with explanatory variables.

The sample is subject to the inverse causality of the explanatory variables towards the dependent one. Under these conditions, methods such as OLS, FE, and RE may return inaccurate estimates, using linear regression techniques. The generalised method of moments (GMM), or instrumental variable panel VAR, may be used to resolve issues such as simultaneity bias, reverse causality or omitted variables. Therefore, GMM is employed as a final stage in the comparative regression analysis, being a dynamic model, which uses a series of instrumental variables, generated from lagged dependent variables.

After testing the linear relationships between variables, revealing the most influential factors for profitability, a new stage of the analysis will be employed, using quadratic regression analysis. The stage actually means that the regression models previously used will be retested adding for every explanatory variable its quadratic form. This will indicate if there is a non-linear effect on the performance of oil and gas companies rather than a straight influence from the variables used to explain changes in ROE.

## Analysis and results

### Correlations between variables

The matrix presented in Table 2 includes the Pearson correlation coefficients between every pair of variables analysed, with the corresponding p-value reflecting whether or not the correlation is statistically significant. The significant correlations (with p-value<0.05) were highlighted in bold in the table.

**Table 2:** Correlations between ROE and indicators with potential impact on profitability.

	ROE	CF/OpRev	CurrRat	AssTn	SolvRat	StockTn	lTax	OilPrice	OilVolume
**ROE**	1								
**CF/OpRev**	**0.2548**	1							
p-value	0								
**CurrRat**	-0.0626	**0.1281**	1						
p-value	0.2973	0.0324							
**AssTn**	**0.1362**	**-0.4957**	**-0.126**	1					
p-value	0.0229	0	0.0354						
**SolvRat**	**-0.2265**	**0.2535**	**0.3466**	**-0.1469**	1				
p-value	0.0001	0	0	0.014					
**StockTn**	**0.148**	-0.0077	**0.2944**	0.0724	-0.0021	1			
p-value	0.0133	0.8976	0	0.2278	0.9715				
**lTax**	-0.0308	**-0.2089**	**-0.1855**	0.0095	**-0.1854**	0.0052	1		
p-value	0.6283	0.0009	0.0032	0.8811	0.0033	0.9349			
**OilPrice**	**-0.1267**	-0.0296	-0.016	0.0029	0.0303	-0.0763	0.0375	1	
p-value	0.0344	0.6222	0.7905	0.9617	0.6139	0.2039	0.555		
**OilVolume**	-0.0631	0.0953	0.0652	0.0302	0.0405	-0.0678	-0.0028	**0.3893**	1
p-value	0.2935	0.1124	0.2778	0.615	0.5002	0.2587	0.9651	0	

It seems that the cash flow over operating revenue ratio, the asset turnover, and stock turnover positively influence the profitability indicator. Moreover, the solvency ratio and oil price also appear to be statistically significant factors, with a negative influence on ROE. The other variables, current ratio, the level of taxation and the volume of oil traded also restrict the return on equity, but their correlation coefficients are small and are not statistically significant, indicating very low to no impact.

The rest of the variables present various significant correlations between each other, but they do not present substantial coefficients, which means that they can be included in the same regression model without implying autocorrelation issues related to independent variables.

Overall, the cash flow over operating revenue ratio is in a direct relationship with the current and solvency ratios, and in a negative relationship with the asset turnover and the level of taxes paid. Moreover, the ability to pay off short-term liabilities with current assets should increase along with the solvency ratio and stock turnover, being affected, by great asset turnover and substantial levels of taxes paid. Results also reveal an indirect relationship between asset turnover and solvency ratio, indicating reduced asset efficiency when oil and gas companies have high debt ratios. Lower taxes are associated with higher solvency ratios, based on lower profits and interest deductibility. Finally, the volume of the oil traded seems to be dependent only on the oil price, following the same trend.

### Linear regression analysis

The comparative regression models were computed, and the main results were included in Table 3. They are robust in term of the coefficient signs and significance, regardless of the static or dynamic models used, showing that the regression model proposed to determine the variance in ROE is correct when we consider the oil and gas companies in the UK.

**Table 3:** Comparative linear regression analysis.

	OLS	FE	RE	FE (corr.)	GMM
L.ROE					0.224***
					(23.91)
L2.ROE					0.146***
					(37.26)
L3.ROE					0.095***
					(18.3)
CF/OpRev	0.953***	0.983***	1.003***	0.957***	1.207***
	(8.36)	(8.40)	(9.07)	(5.01)	(70.43)
CurrRat	-0.162	-0.743	-0.691	-0.551	-0.418
	(-0.26)	(-1.19)	(4.89)	(-0.90)	(-1.12)
AssTn	5.552***	8.826***	7.146***	8.342*	6.148***
	(5.29)	(4.36)	(4.89)	(1.80)	(3.80)
SolvRat	-0.635***	-0.911***	-0.765***	-0.866***	-0.320***
	(-5.82)	(-5.50)	(-5.49)	(-2.85)	(-5.46)
StockTn	0.074**	0.086***	0.087***	0.071**	0.062***
	(2.28)	(3.04)	(3.14)	(3.15)	(3.30)
lTax	0.186	7.645***	3.427*	6.908***	0.418
	(0.17)	(2.98)	1.94	(2.72)	(0.56)
cons	13.259	-67.562**	0.011	-46.962	
	(0.82)	(-2.26)	-0.24	(-1.60)	
R-Squared	0.31	0.38	0.37	0.69	
F / Wald Test	17.82***	22.12***	128.94***	5.64***	91599.84***
Hausman (chi-squared test)			11.35*		
Time fixed effects (F test)		1.86*			
Heteroskedasticity(chi-squared test)		9712.79***			
Sargan (prob.)					21.29 (0.8128)
Arr-Bond test (prob.)					-1.533 (0.13)
					0.757 (0.45)

*p< 0.1,

**p< 0.05,

***p< 0.01;

t statistics are reported in parenthesis; L.ROE, L2.ROE, L3.ROE represents the regression coefficients between the dependent variable, and lagged dependent variable from one, two, and three previous years respectively.

From all the explanatory variables, results show that asset turnover and cash flow over operating revenue have the highest regression coefficients. Based on their values, a unit change in the asset turnover ratio will bring more than five units change in the same direction of the ROE, while one unit change in the cash-flow ratio induces a similar change in ROE. Although the asset turnover coefficient is the highest, the impact on ROE is at a lower level because the average value of the asset turnover (1.45) is 20 times lower than the average ROE (22.67). Along with these two variables, stock turnover also has a significant direct impact on ROE. Based on these positive coefficients, statistically significant, we can say that the profitability of oil and gas companies increases with the level of cash generated and the efficient usage of assets, as well as based on the number of times the inventory is sold or used over a year.

Solvency ratio is also an influential variable, having a negative influence on ROE. This indicates that oil and gas companies in the UK perform better when they dispose of more liabilities (registering a higher degree of solvency). Also, the current ratio also has an indirect impact on profitability, but this is not statistically significant in any of the regression models used. This might also be related to the level of short-term debt, which may affect the general profitability when it increases.

The level of taxation also has a positive effect on ROE, with statistically significant coefficients in all the models besides OLS and GMM. Considering that the taxation variable includes logarithm values of the taxes paid annually by the oil and gas companies, having an average of 11.89 (compared to 27.67 for average ROE), based on the statistically significant regression coefficients of approximately 7, we can can assume that a high level of taxes paid does not affect the oil and gas companies profitability, as they do not face high tax burdens.

The Pooled OLS model applied on return on equity, shows that 31% of the variation in ROE is explained by the independent variables used. The fixed effects (FE), followed by random effects (RE) models, return higher R squared values, indicating that these are better models for the profitability indicator studied. The Hausman test suggests that there are specific characteristics in oil and gas companies, which influence the relationships between variables. There is enough reason for computing a corrected fixed-effect model (denoted “FE (corr.)”), adjusting for time fixed effects and heteroskedasticity to return reliable results.

The final model employed was the Generalized Method of Moments using the lagged dependent variable. It is more performant regarding simultaneity bias, reverse causality, and omitted variables, and thus its results should be the most reliable in this type of analysis. They are also robust, due to the fact that they are similar to the results returned by the static models used. In addition, the profitability variable indicates a dependence on the previous years ROE levels. The Sargan test validates the over-identifying restrictions, with a substantial probability of 81%, while the Arrelano Bond test for serial correlation in the first-differenced errors also validates the GMM models results.

In order to test the robustness again, we include in the model the three control variables related to oil price and volume traded over the period analysed. The comparative regressions results are included in Table 4.

**Table 4:** Comparative linear regression analysis (control variables included in the model).

	OLS	FE	RE	RE (corr.)	GMM
L.ROE					0.201***
					(14.01)
L2.ROE					0.116***
					(5.37)
L3.ROE					0.112***
					(8.70)
CF/OpRev	0.978***	1.003***	1.033***	1.033***	1.078***
	(8.42)	(8.44)	(9.20)	(5.42)	(13.98)
CurrRat	-0.054	-0.596	-0.547	-0.547	-0.665
	(-0.09)	(-0.96)	(-0.91)	(-1.40)	(-2.62)
AssTn	5.764***	9.532***	7.717***	7.717	6.170***
	(5.44)	(4.75)	(5.21)	(1.39)	(4.34)
SolvRat	-0.634***	-0.874***	-0.751***	-0.751***	-0.264***
	(-5.82)	(-5.35)	(-5.39)	(-2.82)	(-3.78)
StockTn	0.066**	0.075***	0.077***	0.077***	0.038***
	(2.04)	(2.67)	(2.79)	(2.60)	(3.21)
lTax	0.298	8.09***	3.942**	3.942*	-0.515
	(0.27)	(3.18)	2.20	1.85	(-0.35)
OilPrice	-0.087	-0.154	-0.131	-0.131	-0.068
	(-0.41)	(-0.98)	(-0.83)	(-1.12)	(-1.12)
OilVolume	-3.186	-2.829*	-2.982**	-2.981	0.155
	(-1.58)	(-1.89)	(-1.99)	(-1.57)	(0.11)
dummyOilPrice	-0.738	-1.073	-0.386	-0.386	-1.702*
	(-0.13)	(-0.26)	(-0.09)	(-0.14)	(-1.66)
cons	34.819	-48.129**	-5.569	-5.569	-12.179
	(1.62)	(-1.56)	(-0.23)	(-0.18)	(-1.23)
R-Squared	0.32	0.41	0.4	0.4	
F / Wald Test	12.46***	16.27***	142.12***	44.53***	146945.8***
Hausman (chi-squared test)			11.43		
Heteroskedasticity (chi-squared test)		13255.25***			
LM Test (chi-squared test)			170.59***		
Sargan (prob.)					19.19 (0.8920)
Arr-Bond test (prob.)					-1.516 (0.13)
					1.004 (0.32)

*p< 0.1,

**p< 0.05,

***p< 0.01;

t statistics are reported in parenthesis; L.ROE, L2.ROE, L3.ROE represents the regression coefficients between the dependent variable, and lagged dependent variable from one, two, and three previous years respectively.

Slight changes appear only in term of the coefficient values, which are a little higher for most variables. However, the statistically significant regression coefficients have the same signs and indicate the same independent variables with a positive influence on ROE regardless of the model used, cash flow/operating revenue, asset turnover, stock turnover, and negative influence from the solvency ratio. The taxation variable returns a significant direct impact only from the fixed and random effect models.

Regarding the control variables, all regression coefficients show a negative impact on ROE. While the results show that oil and gas companies tend to be more profitable when oil price and the volume of the oil traded decrease, this would not be the logic effect. However, we can assume that in the first part of the period analysed, when the crude oil price and volume traded were reduced, the oil and gas companies from the UK were more profitable. As long as the regression coefficients of the control variables are not statistically significant, it means that this assumption cannot be confirmed. However, the GMM results indicate a negative relationship between the dummy and ROE. As the dummy was computed in order to capture the oil price drop (0 for an increase in price, 1 for the decrease in price), this coefficient suggests that over the nine years period, a decrease in oil prices did affect the profitability of oil and gas companies.

The goodness of fit indicator for these models show again a 32% of the variation in ROE explained through the Pooled Ordinary Least Square regression model, and 40% of the variation explained by the fixed effects and random effects models. The Hausman test suggests that in this case, random effects are more appropriate for the oil and gas companies database, indicating that the specific company characteristics do not affect the results. Therefore, a new random effect model corrected for heteroskedasticity was tested. The GMM model shows similar results, including the fact that profitability is directly dependent on the previous years levels, with a Sargan test which validates the over-identifying restrictions with a probability of 89%. The Arellano Bond test for serial correlation in the first-differenced errors also confirms the GMM models results.

### Descriptive analysis

**Fig 1:**
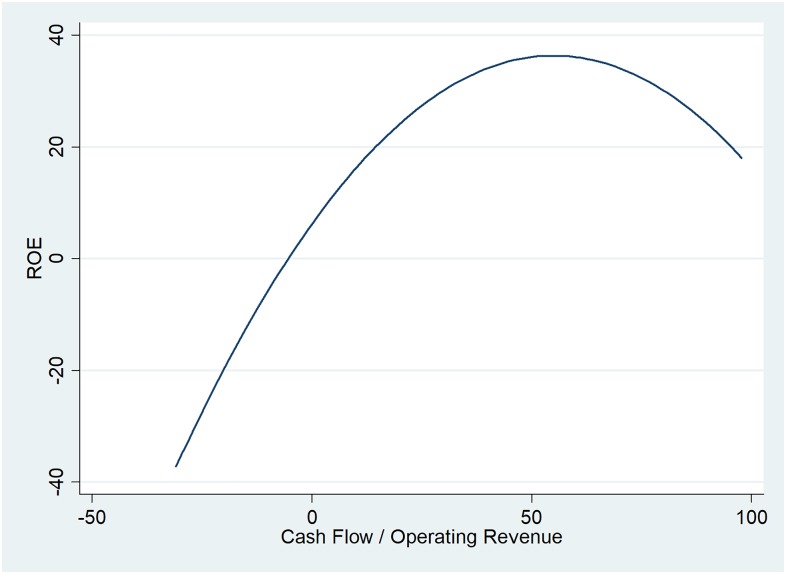
Two-way quadratic prediction plot between ROE and cash flow over operating revenue ratio.

The cash flow over operating revenue ratio is a complex indicator as it refers to more than company sales of goods and services. This ratio offers an overview of the financial health of the company, the sales effectiveness, its liquidity and cash management. Therefore, higher ratios reflect companies’ ability to generate cash from its sales. For the oil and gas companies in the UK, it appears that the return on equity increases along with the cash flows from operating activities, but for a ratio up to 50%. The figure below (Fig 1) indicates that non-profitable companies register negative cash flows. However, this is just a singular case for eight of the companies included in the database, managing to recover the following year.

**Fig 2:**
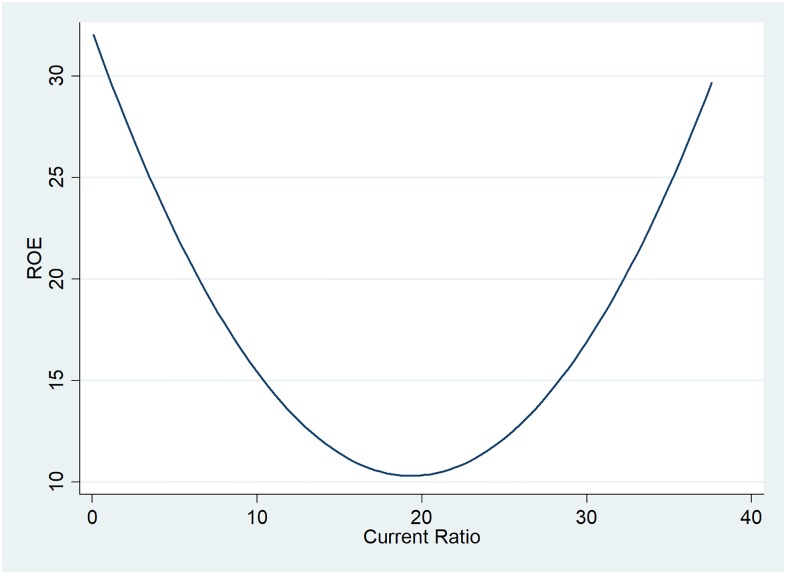
Two-way quadratic prediction plot between ROE and current ratio.

For the companies analysed, there are minor differences between the current ratio and liquidity ratio. Therefore we chose the first, used to measure the firm’s ability to pay off its short-term liabilities with its current assets. As observed in Fig 2, increased profitability is associated with low current ratios, declining along with an increase in the level of current ratio, of up to 20. For current ratios higher than 20, ROE is positively influenced, increasing as well. However, this is an exception, as in 95% of the data analysed the current ratio is of maximum 7.

**Fig 3:**
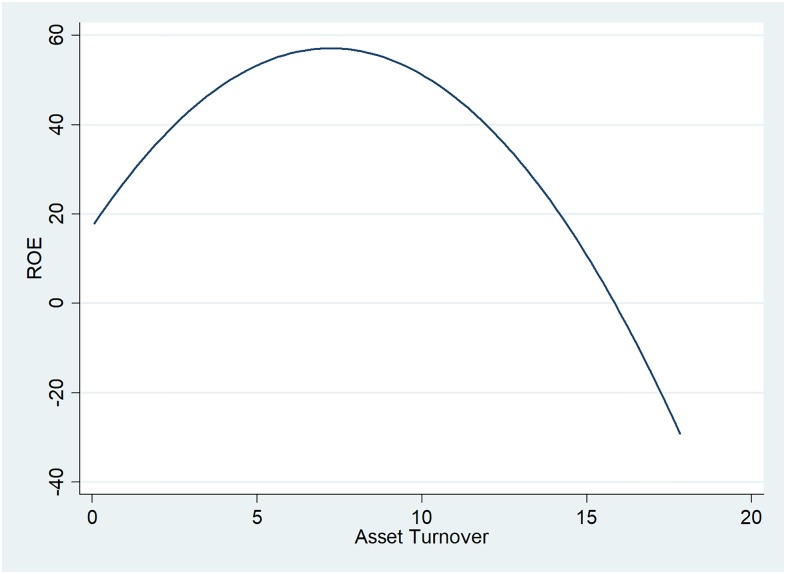
Two-way quadratic prediction plot between ROE and asset turnover.

As seen in Fig 3, there is a direct relationship between ROE and asset turnover, indicating that profitable companies efficiently use their assets in generating sales and other types of revenues. It is the ideal nexus between the two indicators, as oil and gas companies have fixed assets of high values as long as significant short-term items such as inventory, indicating that they benefit from economies of scale but also maintain their equipment and machinery in order to minimise downtime. For asset turnover ratios higher than 7, companies tend to become less profitable, this being the case in only 3% of the data analysed.

**Fig 4:**
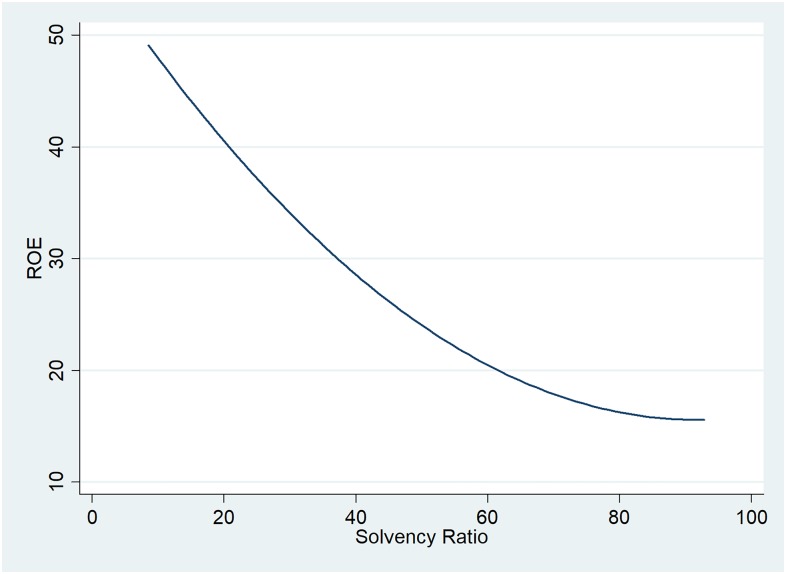
Two-way quadratic prediction plot between ROE and solvency ratio.

Fig 4 indicates a solely indirect relationship between ROE and solvency ratio, showing that oil and gas companies in the UK are more profitable when they register lower solvency ratios, although these would generally be associated with a higher probability for companies to face default on their debt obligations. The companies analysed record high profit margins, and thus when they dispose of more borrowed funds they can increase their net profits.

**Fig 5:**
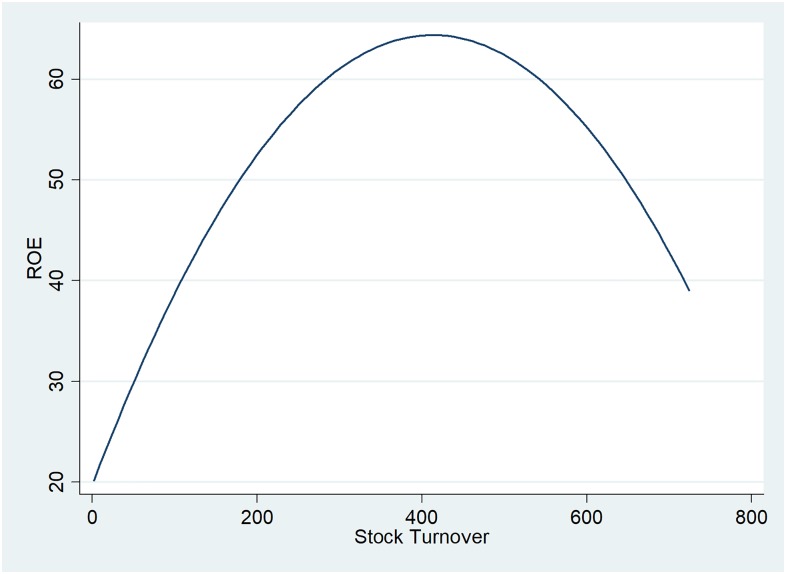
Two-way quadratic prediction plot between ROE and stock turnover.

The stock turnover, reflecting the number of times the inventory is sold or used over a period of one year, is very high for the UK oil and gas companies, indicating operational efficiency in the asset management department. Fig 5 suggests a direct relationship between profitability and stock turnover, up to a peak stock turnover of 400, which was found only in two cases in the overall data, showing the exceptional character of the negative impact of stock turnover on ROE.

**Fig 6:**
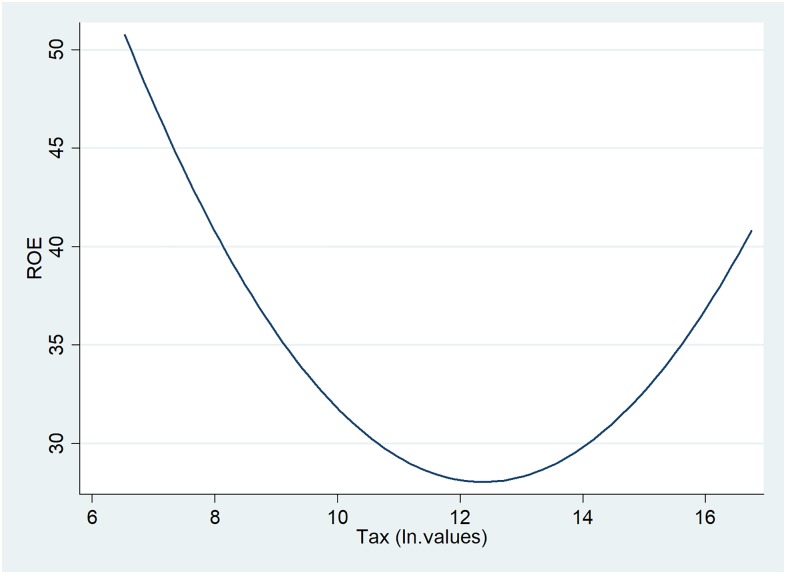
Two-way quadratic prediction plot between ROE and tax.

For the oil and gas companies analysed, more than 50% pay taxes higher than 100 million Euros per year. Fig 6 illustrates the relationship between profitability and taxation, showing a structural break at a level of taxation of approximately 160 millions of Euros (value 12 on the x-axis). From that level onward, companies tend to be more profitable when they pay higher taxes.

**Fig 7:**
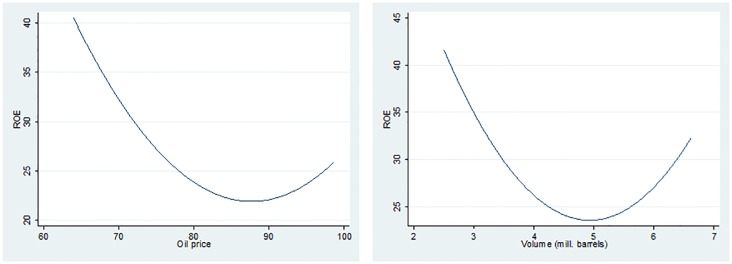
Two-way quadratic prediction plot between ROE and oil price, and volume of oil.

Over the period analysed, the oil price had an average price ranging from 64 $/barrel to a little below 100 $/barrel. The first part of the period indicated lower prices, with the peak in 2008 (98.58 $/barrel), while from 2011 the price oscillated above 90 $/barrel. Considering this information, it can be presumed from the graph illustrated in Fig 7 that the UK oil and gas companies were the most profitable in the first part of the period analysed, before the settlement and deepening of the financial crisis, indicating larger values of ROE in spite of lower oil prices. The average oil volume traded monthly over the period analysed ranged from 2.5 to 6.6 millions of barrels. Although the smallest volume traded was registered in 2006, the oil and gas companies in the UK were the most profitable in term of shareholders’ equity. It confirms the assumptions mentioned based on the dynamics of oil prices. After 2011, the volume traded decreased from an average of 6.5 millions of barrels to approximately 5 million, affecting the profitability of oil and gas companies, as shown in the second graph from Fig 7, illustrating the relationship between ROE and volume of oil traded in the UK.

### Quadratic regression analysis

Observing from the descriptive analysis that the independent variables, except the solvency ratio, have a non-linear relationship with companies profitability, we decided to retest the regression model, including quadratic variables as well. We assume that the relationships discovered through linear regression analysis are the main ones, and the different influences occur in exceptional cases, associated with the squared variables. Results are presented in the following table (Table 5).

**Table 5:** Comparative quadratic regression analysis.

	OLS	FE	RE	FE (corr.)	GMM
L.ROE					0.107***
					(4.92)
L2.ROE					0.085***
					(4.94)
L3.ROE					0.053**
					(2.38)
CF/OpRev	1.783***	0.976***	1.371***	0.976**	2.074***
	(8.20)	(4.78)	(6.55)	(2.40)	(27.56)
CF/OpRev sq.	-0.009***	-0.0004	-0.004	-0.0004	-0.012***
	(-2.94)	(-0.17)	(-1.3)	(-0.14)	(-15.64)
CurrRat	1.029	1.593	0.819	1.593	2.964***
	(0.87)	(1.23)	(0.66)	(1.27)	(6.32)
CurrRat sq.	-0.032	-0.051	-0.033	-0.051*	-0.073***
	(-0.86)	(-1.44)	(-0.93)	(-1.97)	(-6.60)
AssTn	22.82***	33.247***	22.038***	33.247***	31.65***
	(10.39)	(8.88)	(8.97)	(3.18)	(7.97)
AssTn sq.	-1.288***	-1.443***	-1.154***	-1.443***	-1.595***
	(-8.3)	(-7.74)	(-7.15)	(-3.44)	(-8.48)
SolvRat	-1.453***	-1.629***	-1.603***	-1.629*	-0.982**
	(-3.57)	(-3.62)	(-3.74)	(-1.88)	(-2.47)
SolvRat sq.	0.008**	0.007	0.009**	0.007	0.001
	(2.04)	(1.41)	(2.06)	(0.85)	(0.23)
StockTn	0.155**	0.137**	0.185***	0.137**	0.048
	(2.56)	(2.08)	(2.93)	(2.30)	(1.04)
StockTn sq.	-0.0002**	-0.0001	-0.0002**	-0.0001**	-0.0001**
	(-2.25)	(-1.29)	(-2.25)	(-2.06)	(-1.96)
lTax	-21.883***	-15.103	-17.293*	-15.103	-11.906*
	(-2.76)	(-1.13)	(-1.74)	(-1.26)	(-1.71)
lTax sq.	0.937***	0.879	0.794*	0.879	0.674**
	(2.82)	(1.47)	(1.89)	(1.44)	(2.21)
cons	13.259	58.392	97.162	58.392	19.415
	(0.82)	(0.76)	(1.63)	(0.93)	(0.49)
R-Squared	0.52	0.55	0.48	0.55	
F / Wald Test	21.23***	20.77***	220.76***	25.91***	121082.04***
Hausman (chi-squared test)			58.19***		
Time fixed effects (F test)		0.92			
Heteroskedasticity (chi-squared test)		6421.69***			
Sargan (prob.)					13.53 (0.9902)
Arr-Bond test (prob.)					-1.803 (0.07)
					-0.214 (0.83)

*p< 0.1,

**p< 0.05,

***p< 0.01;

t statistics are reported in parenthesis; L.ROE, L2.ROE, L3.ROE represents the regression coefficients between the dependent variable, and lagged dependent variable from one, two, and three previous years respectively.

Results show that cash flow over operating revenue has a positive influence on ROE, but from a certain point (from a ratio of 50 as suggested in Fig 1), it affects profitability to a very small extent. It is consistent with the results offered by the linear regressions.

The current ratio indicates a positive influence and a negative one from the squared ratio. The graph presented in Fig 2 indicated a U-shape, suggesting a negative relationship followed by a direct one for current ratios higher than 20. The current ratio regression coefficients cannot be considered a relevant ROE factor, as they are statistically significant only in the GMM model.

Asset turnover coefficients follow the trend reflected in Fig 3: this indicator has a strong direct influence on ROE (with large values of regression coefficient), but for asset turnover ratios higher than seven they start to affect the companies’ profitability (as shown by the squared variable coefficient).

Solvency ratio only has a statistically significant indirect impact on ROE, as shown in Fig 4, confirming the fact that oil and gas companies with significant levels of liabilities tend to be more profitable. The squared variable has a positive influence, but a low impact on ROE, as suggested by the almost null regression coefficient.

The stock turnover confirms the inverted U-shape presented in Fig 5. Efficient companies regarding the asset management department tend to be more profitable, but up to a point, where significant stock turnover tends to affect the companies return on equity. The negative coefficient is very low, being attributed to exceptional cases of high stock turnover.

Taxation variables also follow the trend presented in the descriptive analysis: for taxes up to 160 millions of Euros (see comments on Fig 6), companies profitability is affected by the tremendous level of expenses on taxes; but for companies registering even higher tax payments, the returns on equity is no longer concerned, as tax burdens do not raise based on the increase in revenues. However, tax variables coefficients are statistically significant only in the OLS and GMM models.

This quadratic regression model indicates higher goodness of fit indicators. The Pooled Ordinary Least Square regression model surprises 52% of the variation in ROE, increasing to 55% of the variation explained through the fixed effects model. The Hausman test suggests that this is more appropriate for the oil and gas companies’ database, as specific company characteristics affect the results. The GMM model is the best employed so far, with a Sargan test validating the over-identifying restrictions with a probability of 99%, and the serial correlation in the first-differenced errors confirming the GMM results.

Considering that the oil price and oil volume variables also revealed a non-linear relationship with ROE, we retested the quadratic regression model, including the three control variables, even with the squared variables of oil price and oil volume. There are no significant changes in the results (Table 6), proving their robustness: the cash flow over operating revenue, current ratio, asset turnover, and stock turnover have a positive and then negative influence on ROE, while the tax and solvency ratio indicates a negative impact followed by a positive one. From all independent variables, current ratio shows statistically significant coefficients only from GMM, taxation variables are significant in OLS and GMM, and solvency ratio appears to have only a statistically significant negative influence on profitability.

**Table 6:** Comparative quadratic regression analysis (control variables included in the model).

	OLS	FE	RE	FE (corr.)	GMM
L.ROE					0.108**
					(2.08)
CF/OpRev	1.824***	1.016***	1.379***	1.016**	1.896***
	(8.30)	(4.93)	(6.57)	(2.58)	(12.36)
CF/OpRev sq.	-0.009***	0.0005	-0.004	0.00005	-0.010***
	(-3.01)	(0.02)	(-1.28)	(0.02)	(-9.66)
CurrRat	1.044	1.664	0.846	1.664	3.833**
	(0.87)	(1.27)	(0.68)	(1.26)	(2.01)
CurrRat sq.	-0.029	-0.05	-0.032	-0.05*	-0.099**
	(-0.78)	(-1.39)	(-0.87)	(-1.76)	(-2.13)
AssTn	22.84***	33.382***	22.139***	33.382***	36.249***
	(10.29)	(8.83)	(8.75)	(3.15)	(6.50)
AssTn sq.	-1.280***	-1.415***	-1.129***	-1.415***	-1.476***
	(-8.18)	(-7.53)	(-6.91)	(-3.35)	(-6.44)
SolvRat	-1.417***	-1.589***	-1.564***	-1.589*	-1.185**
	(-3.47)	(-3.52)	(-3.62)	(-1.87)	(-2.17)
SolvRat sq.	0.008*	0.007	0.009**	0.007	0.005
	(1.94)	(1.40)	(1.98)	(0.84)	(0.95)
StockTn	0.135**	0.102	0.157**	0.102	0.081*
	(2.20)	(1.49)	(2.42)	(1.51)	(1.83)
StockTn sq.	-0.0002**	-0.0001	-0.0002**	-0.0001	-0.00005
	(-1.97)	(-0.80)	(-1.82)	(-1.146)	(-1.35)
lTax	-21.292***	-12.101	-15.043	-12.1	2.989
	(-2.68)	(-0.88)	(-1.46)	(-1.01)	(0.14)
lTax sq.	0.914***	0.761	0.709*	0.762	0.067
	(2.74)	(1.24)	(1.69)	(1.25)	(0.06)
OilPrice	-1.313	-0.362	-1.43	-0.362	-1.906*
	(-0.46)	(-0.16)	(-0.59)	(-0.19)	(-1.69)
OilPrice sq.	0.008	0.002	0.008	0.002	0.011
	(0.44)	(0.13)	(0.56)	(0.16)	(1.56)
OilVolume	-10.774	-3.095	-10.163	-3.095	12.466
	(-1.01)	(-0.37)	(-1.13)	(-0.5)	(0.38)
OilVolume sq.	0.919	0.099	0.879	0.099	-1.102
	(0.80)	(0.11)	(0.90)	(0.17)	(-0.37)
dummyOilPrice	3.405	-1.029	2.471	-1.03	-1.675
	(0.67)	(-0.26)	(0.58)	(-0.31)	(-1.16)
cons	200.118	69.269	169.439	69.269	
	(1.57)	(0.57)	(1.44)	(0.65)	
R-Squared	0.53	0.56	0.499	0.56	
F / Wald Test	15.18***	15.04***	226.11***	29.59***	48995.35***
Hausman (chi-squared test)			43.48***		
Time fixed effects (F test)		0.46			
Heteroskedasticity (chi-squared test)		6421.69***			
Sargan (prob.)					13.18 (0.9995)
Arr-Bond test (prob.)					-2.545 (0.01)
					-0.59 (0.55)

*p< 0.1,

**p< 0.05,

***p< 0.01;

t statistics are reported in parenthesis; L.ROE represents the regression coefficient between the dependent variable, and lagged dependent variable from the previous year.

The control variables do not reveal statistically significant regression coefficients, except in the GMM case, where the basic variables of the oil price have a negative influence on ROE, showing that lower prices of crude oil are associated to higher profitability over the period analysed.

The goodness of fit indicators for this updated quadratic regression model are slightly higher than the rest, showing that the Pooled Ordinary Least Square regression model surprises 53% of the variation in ROE, increasing to 56% of the variation explained through the fixed effects model. The Hausman test suggests again that company characteristics affect the results and the fixed effects model is more appropriate. In addition, Sargan test attributed to the GMM validates the over-identifying restrictions with a probability of 99.95%.

According to the homogeneous values of the data analysed, the profitability of oil and gas companies increases with the level of cash generated and the efficient usage of assets, as well as based on the number of times the inventory is sold or used over a year. These show a productive operational activity for oil and gas companies in the UK. Correspondingly, these tend to perform better when they dispose of more liabilities. While the oil price changes and oil volume traded does not seem to have a substantial impact on the analysed companies, results suggest that a decrease in oil prices affects their profitability. Although it seems that companies easily pass the oil price increases on to customers, if the oil price continues to drop, it would permanently affect both the oil and gas companies and the economy, as long as oil and gas industry is still an important pillar for the UK economy, bringing significant tax revenues.

## Conclusions

As national strategic resources, the North Sea oil and gas is essential to the economy and development of the United Kingdom. With large energy companies operating, two of the six supermajor oil and gas companies have their headquarters in the UK: BP and Royal Dutch Shell. However, since the financial crisis started, due to the oil price volatility, the employment in the UK offshore oil and gas industry was reduced with 25% over the last five years, while oil and gas companies reduced their activity.

Although lately, they faced the challenges of a volatile environment with a reduction in production, oil and gas companies managed to recover and continue to register large profit margins and returns. The high-performance organisation is obtainable through operational efficiency in the asset management department, efficiently use of assets in generating sales, and ability to permanently generate cash from sales. Oil and gas companies seem to be performant in the use of liabilities, with significant levels of solvency, and the higher the amount of taxes paid are, the more profitable regarding shareholders’ equity they tend to be. It also shows that the oil and gas industry continues to be an essential pillar in the UK economy, providing substantial fiscal revenues based on profitable oil and gas companies.

The latest dropout in oil price and volume traded affected the return on equity, as oil and gas companies indicated higher profitability at the beginning of the period analysed when, although the oil price and volume traded were at their minimum over the nine years observed, the companies registered the highest returns and profit margins. If the oil price reduction continues, this will continue to affect oil and gas companies performance, along with the UK economy, through increased unemployment and reduced taxes paid to the government, towards a permanent decrease in GDP.

Problems related to various impacts of the global financial crisis permanently challenge the oil and gas companies, to cope with the decrease in oil price and the changes in production and revenues. Therefore, the influence of unobserved heterogeneity in the parameters used in this paper, and the ways to conduct quantitative analysis to observe these influential factors were exemplified in different stages of the analysis. On the one hand, results indicate that there is unobserved heterogeneity in the sample consisting of 31 oil companies, and therefore, estimation techniques employed may produce biased efficiency estimates. On the other hand, results demonstrated their robustness through different regression models and techniques, proving the consistency of this research.

Nowadays, the largest oil and gas companies seem to take the climate commitment very seriously and pledge to reduce the net carbon emission in the following decades. The latest requirements demand that companies disclose the climate change risks on their business, so the shareholders understand the implications of company activities. Although their shareholders, as well as consumers, approve the change from fossil fuels, oil and gas companies seem to undertake small investments in renewable energy compared to their overall revenues. Overall, considering the importance of renewable energy stocks in the future, we realise that increased oil prices and high prices for other types of fossil fuels will just increase the demand for clean energy products. Even though oil and gas companies seem to have steady profits and returns, over the long-term, the only solution for them to keep their high-performance is switching towards eco-friendly policies.

## References

[1] Sun C, Luo Y, Huang Y, Ouyang X. A comparative study on the production efficiencies of China’s oil companies: A true fixed effect model considering the unobserved heterogeneity. Journal of Cleaner Production. 2017;154: 341–352.

[2] Zhou DQ, Wu F, Zhou P. Output-specific energy efficiency assessment: A data envelopment analysis approach. Applied Energy. 2016;177: 117–126.

[3] Thomsen S, Pedersen T. Ownership structure and economic performance in the largest European companies. Strategic Management Journal. 2000;21: 689–705.

[4] Gompers PA, Ishii JL, Metrick A. Corporate governance and equity prices. Quarterly Journal of Economics. 2003;118: 107–155.

[5] Wolf C. Does ownership matter? The performance and efficiency of State Oil vs Private Oil (1987–2006). Energy Policy. 2009;37: 2642–2652.

[6] Kang W, Perez de Gracia F, Ratti RA. Oil price shocks, policy uncertainty, and stock returns of oil and gas corporations. Journal of International Money and Finance. 2017;70: 344–359.

[7] Diaz E, Perez de Gracia F. Oil price shocks and stock returns of oil and gas corporations. Finance Research Letters. 2017;20: 75–80.

[8] Liu J, Klinkowska O. Impact of oil price changes on stock returns of UK oil and gas companies: A wavelet-based analysis. 2017 https://ssrn.com/abstract=2997025.

[9] Van Alstine J, Manyindo J, Smith L, Dixon J, Amaniga Ruhanga I. Resource governance dynamics: The challenge of ‘new oil’ in Uganda. Resources Policy. 2014;40: 48–58.

[10] EITI-Extractive Industries Transparency Initiative Website. EITI Secretariat, Oslo. 2017. https://eiti.org/about/how-we-work

[11] Sovacool BK, Walter G, Van de Graaf T, Andrews N. Energy governance, transnational rules, and the resource curse: Exploring the effectiveness of the Extractive Industries Transparency Initiative (EITI). World Development. 2016;83: 179–192.

[12] Corduneanu C, Moldovan N, Lobont O. Financial instruments designed to increase the competitiveness of the European firms. EuroEconomica. 2010;3(26): 51–59.

[13] Dayanandan A, Donker H. Oil prices and accounting profits of oil and gas companies. International Review of Financial Analysis. 2011;20: 252–257.

[14] Ramos SB, Veiga H. Risk factors in oil and gas industry returns: International evidence. Energy Economics. 2011;33: 525–542.

[15] Banerjee SB. Corporate citizenship and indigenous stakeholders: Exploring a new dynamic of organisational stakeholder relationships. Journal of Corporate Citizenship. 2001;1(1): 39–55.

[16] Porter ME, Kramer MR. The link between competitive advantage and corporate social responsibility. Harvard Business Review. 2006;84(6): 1–14. 17183795

[17] Brindescu-Olariu D. Assessment of the bankruptcy risk based on the solvency ratio. Theoretical and Applied Economics. 2016;3(608): 257–266.

[18] Asuquo AI. Environmental friendly policies and their financial effects on corporate performance of selected oil and gas companies in Niger Delta region of Nigeria. American International Journal of Contemporary Research. 2012;2(1): 168–173.

